# Vaccine effect of recombinant single-chain hemagglutinin protein as an antigen

**DOI:** 10.1016/j.heliyon.2020.e04301

**Published:** 2020-06-27

**Authors:** Atsushi Kawai, Yasuyuki Yamamoto, Yasuo Yoshioka

**Affiliations:** a Laboratory of Nano-design for Innovative Drug Development, Graduate School of Pharmaceutical Sciences, Osaka University, 1-6 Yamadaoka, Suita, Osaka 565-0871, Japan; b Vaccine Creation Group, BIKEN Innovative Vaccine Research Alliance Laboratories, Research Institute for Microbial Diseases, Osaka University, 3-1 Yamadaoka, Suita, Osaka 565-0871, Japan; c Vaccine Creation Group, BIKEN Innovative Vaccine Research Alliance Laboratories, Institute for Open and Transdisciplinary Research Initiatives, Osaka University, 3-1 Yamadaoka, Suita, Osaka 565-0871, Japan; d BIKEN Center for Innovative Vaccine Research and Development, The Research Foundation for Microbial Diseases of Osaka University, 3-1 Yamadaoka, Suita, Osaka 565-0871, Japan; e Global Center for Medical Engineering and Informatics, Osaka University, 3-1 Yamadaoka, Suita, Osaka 565-0871, Japan

**Keywords:** Immunology, Microbiology, Virology, Proteins, Pharmaceutical science, Infectious disease, Pharmacology, Adjuvant, Hemagglutinin, Influenza virus, Recombinant protein, Vaccine

## Abstract

Vaccination is one of the most effective interventions for preventing the spread of influenza viruses at the population level. Currently most influenza vaccines are produced by using embryonated chicken eggs, but alternative methods that achieve more rapid large-scale production are highly desirable for vaccines against both pandemic and seasonal influenza viruses. The use of recombinant hemagglutinin (HA), a key virus surface protein, as an antigen is an attractive candidate alternative approach, because of the potential for high protein yields and the ease of cloning new antigenic variants. Although fusion of HA with trimerization domains is needed to stabilize the trimeric structure and enhance the immunogenicity of the recombinant HA protein, whether the trimerization domains are immunogenic must be considered. Here, we generated recombinant multimeric HA without trimerization domains by using a short peptide linker, termed a single-chain HA (scHA), and evaluated scHAs as potential antigens for generating vaccines against influenza virus. Using mammalian cells, we succeeded in making three types of recombinant scHAs—two dimeric scHAs and a trimeric scHA. After immunization with aluminium salts in mice, one of the dimeric scHAs induced the greatest HA-specific IgG response among the scHAs and protected against virus challenge as strongly as the typically used trimeric HA containing a trimerization domain. We did not observe IgGs specific for the short peptide linker in mice immunized with the dimeric scHA, although IgGs specific for the trimerization domain occurred in mice immunized with the trimeric HA containing that domain. Furthermore, changing to another adjuvant did not diminish the utility of the dimeric scHA. These results suggest the potential usefulness of dimeric scHA as a vaccine antigen. We believe that single-chain antigens may represent new alternatives for production of recombinant antigen–based vaccines.

## 1 Introduction

Influenza A viruses remain a serious public health threat worldwide. According to the World Health Organization (https://www.who.int/en/news-room/fact-sheets/detail/influenza-(seasonal)), seasonal influenza viruses cause approximately 290,000–650,000 deaths annually worldwide, particularly in young and elderly people. Almost all current influenza vaccines confer potent protection against antigenically matched or closely related homologous viruses infections by inducing strain-specific antibodies to key virus surface proteins, such as hemagglutinin (HA) [1]. However, these strain-specific antibodies cannot protect against antigenically drifted heterologous viruses, due to mutation of the HA receptor-binding site [2, 3]. In fact, heterologous viruses contributed to the significantly decreased vaccine effectiveness—as low as 19%—that was reported during the 2014–2015 season [4], although meta-analysis of clinical studies over several influenza seasons indicates an efficacy of approximately 60% in adults against seasonal influenza [5]. In addition to seasonal heterologous strains, pandemic strains such as the antigenically divergent H5N1 and H7N9 avian influenza viruses are extremely worrisome from a public health standpoint, because of the ineffectiveness of seasonal vaccines against these pandemic strains. For example, avian-to-human transmission of H7N9 influenza virus in China led to several hundred cases of flu in humans, with a case fatality rate of approximately 40% [6].

Currently, most influenza vaccines are produced by using embryonated chicken eggs. When a pandemic strain emerges, it takes approximately 6 months to produce and distribute enough new vaccine to protect a substantial proportion of the population [2], in part due to the time needed to generate sufficient pathogen-free eggs and to adapt the virus to grow within eggs. This lag means there is insufficient vaccine available to provide effective protection during the first pandemic wave. In addition, mutations within the HA gene are often induced through adaptation of the virus to eggs, leading to ineffectiveness of the vaccine. Therefore, alternative methods that support more rapid and large-scale production are highly desired for generating vaccines against pandemic as well as seasonal influenza viruses.

The use of recombinant proteins as vaccine antigens is an attractive alternative approach to egg-based vaccine production. This alternative system has several advantages, including no need to handle potentially dangerous live viruses, no need for eggs, negligible chance of mutation within the target-expressed gene, easy upscaling of production, and rapid production of new vaccines; these advantages make this new method ideally suited for pandemic influenza vaccines. For vaccines against new seasonal outbreaks or pandemics, the HA gene in circulating viruses can be cloned directly from clinical isolates, and recombinant protein can be rapidly produced. In addition, many studies indicate the potential of the recombinant ectodomain of HA proteins produced in mammalian and insect cell systems as an antigen [7, 8, 9, 10]. The immunogenicity of the recombinant ectodomain of HA proteins is enhanced through fusion with trimerization domains, to stabilize the native trimeric conformation of HA [7, 10]. Many reports have shown the usefulness of trimerization domains—such as foldon of fibritin from bacteriophage T4 and the isoleucine zipper of the GCN4 transcriptional activator from *Saccharomyces cerevisiae*—in the generation of recombinant HA proteins [7, 8, 9, 10]. However, whether trimerization domains derived from species other than human would be immunogenic when used in recombinant HAs for future clinical applications is unknown. In this regard, vaccination of antigens generated from human immunodeficiency virus and combined with these domains induces antibodies against trimerization domains [11]. Whether these extraneous antibodies influence the vaccine effect of the recombinant antigen is unknown; they might decrease the vaccine's efficacy through the formation of immune complexes between the antigen and antibodies against trimerization domains. In addition, the introduction of immunodominant epitopes on a trimerization domain might act as a decoy, diverting antibody responses to vaccine antigens and decreasing vaccine efficacy. Furthermore, detailed evaluation of the cross-reactivity of these antibodies against trimerization domains to human endogenous proteins is needed to avoid severe adverse effects. Therefore, a novel platform for generating recombinant HAs without trimerization domains is urgently needed.

Here, we generated recombinant multimeric HA that lack the trimerization domain by using short peptide linkers, termed single-chain HA (scHA), and examined the potential of several types of scHAs as antigens in vaccines against influenza virus. We showed that dimeric scHA induces an HA-specific antibody response that is as strong as that from traditional trimeric recombinant HA that contains the trimerization domain. These data indicate the usefulness of scHAs for increasing the antigenicity of recombinant HA despite the lack of trimerization domains.

## 2 Materials and methods

### 2.1 Reagents

Horseradish-peroxidase–conjugated goat anti-mouse IgG was purchased from Merck Millipore (Darmstadt, Germany). Alhydrogel adjuvant (2%) was purchased from InvivoGen (San Diego, CA, USA). K3 CpG oligodeoxynucleotide (ODN; 5′-atcgactctcgagcgttctc-3′) was purchased from Gene Design (Osaka, Japan). H1N1 influenza A virus (strain: A/California/7/2009 [Cal7]) was kindly provided by Dr. Hideki Asanuma (National Institute of Infectious Diseases, Tokyo, Japan).

### 2.2 Construction of recombinant HA and ovalbumin-expressing vectors

The amino-acid sequence for HA used in this study was derived from A/California/07/2009 (H1N1) (GenBank accession number: ACV82259.1). A human codon–optimized cDNA of the ectodomain of HA (amino acids 1–523) with a C-terminal hexahistidine tag (His-tag) was cloned into the pcDNA3.1 expression plasmid (Thermo Fisher Scientific, Hampton, NH, USA). For trimeric recombinant HA with foldon, the codon-optimized foldon sequence (GYIPEAPRDGQAYVRKDGEWVLLSTFL) was inserted at the C-terminus of HA (amino acids 1–520). For dimeric and trimeric scHAs, monomeric HAs were fused together by using codon-optimized linkers (GGGGS or GGGGSGGGGSGGGGS). In addition, an ovalbumin cDNA with foldon at its C-terminus was cloned into the pcDNA3.1 expression plasmid. For recombinant ovalbumin with linker, an ovalbumin cDNA with codon-optimized linker (GGGGSGGGGSGGGGS) at its C-terminus was cloned into the pcDNA3.1 expression plasmid.

### 2.3 Expression and purification of recombinant HA

All secreted soluble recombinant HAs and recombinant ovalbumins were expressed by using the Expi293 Expression System (Thermo Fisher Scientific) according to the manufacturer's instructions. Briefly, Expi293F cells grown in Expi293 expression medium in Erlenmeyer flasks were diluted to 3.0 × 10^6^ cells/mL. Then 30 μg of each HA- or ovalbumin-expressing plasmid was transfected into 7.5 × 10^7^ Expi293F cells in 30 mL medium by using ExpiFectamine 293 Reagent. Cells were incubated at 37 °C, 8% CO_2_ on an orbital shaker (120 rpm) for 18 h; ExpiFectamine 293 Transfection enhancer 1 and 2 were added and the cells continued incubation at the same conditions. Five days later, the culture medium was collected by centrifugation at 5,000 *g* for 20 min; the recombinant HAs and ovalbumins in the supernatant were then purified by using an Akta explorer chromatography system with Ni-Sepharose HisTrap FF column (GE Healthcare, Diegem, Belgium). Bound recombinant protein was eluted by using 20 mM sodium phosphate buffer (pH 7.4) containing 500 mM imidazole. Recombinant protein in the eluates was further purified by using an Akta explorer chromatography system with a Superose 6 Increase 10/300 GL column (GE Healthcare). A high molecular weight standard kit (Bio-Rad, Hercules, CA, USA) was used to estimate recombinant protein size. Fractions of interest were pooled and then concentrated by using Amicon Ultra centrifugal filter (cut-off, 10 kDa; Merck Millipore). For sodium dodecyl sulfate–polyacrylamide gel electrophoresis (SDS-PAGE), purified proteins were mixed 1:1 (v/v) in sample buffer solution (Nacalai Tesque, Kyoto, Japan) containing 2-mercaptoethanol (Sigma-Aldrich, St. Louis, MO, USA) and heated at 95 °C for 5 min before being loaded onto a 4%–15% Mini-PROTEAN TGX Precast Protein Gel (Bio-Rad). After electrophoresis, gels were stained with Coomassie Brilliant Blue according to standard protocols.

### 2.4 Mice

C57BL/6J male mice were purchased from SLC (Hamamatsu, Japan); they were housed under a 12:12-h light:dark cycle (lights on, 8:00 am) and had unrestricted access to food and water. Mice were used at 6–7 weeks of age. The anesthetic was prepared as a mixture of three drugs: medetomidine (Domitor; Nippon Zenyaku Kogyo Co., Ltd., Fukushima, Japan), midazolam (Dormicum; Maruishi Pharma Inc., Osaka, Japan), and butorphanol (Vetorphale; Meiji Seika Pharma, Ltd., Tokyo, Japan). We mixed together 0.75 mL Domitor, 2 mL Dormicum, 2.5 mL Vetorphale, and 19.75 mL sterilized distilled water, and intraperitoneally injected the solution at 0.1 mL per 10 g of mouse body weight to induce anesthesia. All animal experiments were performed in accordance with Osaka University's institutional guidelines for the ethical treatment of animals and were approved by the Animal Care and Use Committee of the Research Institute for Microbial Diseases, Osaka University, Japan (protocol number, BIKEN-AP-H26-11-0).

### 2.5 Vaccine effect against influenza virus

C57BL/6J naive mice were immunized subcutaneously at the base of the tail on days 0 and 21 by using recombinant HAs (1 μg/mouse) with Alhydrogel adjuvant (250 μg/mouse) or CpG ODN (50 μg/mouse). As a control group, naive mice were immunized subcutaneously with PBS. On day 28, we obtained plasma samples and used ELISA to determine the levels of antibodies specific for monomeric HA, trimeric HA with foldon, influenza A virus (Cal7), or ovalbumins in the plasma. To this end, ELISA plates (Corning, Corning, NY, USA) were coated with monomeric HA (1 μg/mL), trimeric HA with foldon (1 μg/mL), ovalbumin with foldon (1 μg/mL), or ovalbumin with linker (1 μg/mL) in 0.1 M sodium carbonate buffer (pH 9.6) or with Cal7 (1 μg/mL) in PBS by incubating overnight at 4 °C. The coated plates were then incubated with 1% Block Ace (DS Pharma Biomedical, Osaka, Japan) for 1 h at room temperature. Plasma samples were serially diluted by using 0.4% Block Ace, added to the antigen-coated plates, and incubated for 2 h at room temperature; the coated plates were then incubated with horseradish-peroxidase–conjugated goat anti-mouse IgG for 1 h at room temperature. After the incubation, the color reaction was developed by using tetramethyl benzidine (Nacalai Tesque, Kyoto, Japan), stopped with 2 N H_2_SO_4_, and measured at OD_450–570_ on a microplate reader (Power Wave HT, BioTek, Winooski, VT, USA). On day 31, mice were anesthetized and then challenged intranasally with 9 × 10^4^ TCID_50_ (10 × LD_50_) of Cal7 in 30 μL of PBS. Body weights and survival rates of challenged mice were monitored. The humane endpoint was set at 25% loss of body weight relative to the body weight at the time of infection.

### 2.6 Statistical analyses

Statistical analyses were performed by using Prism (GraphPad Software, San Diego, CA, USA). All data are presented as means ±1 standard deviation (SD). ANOVA followed by Tukey's test was used to determine significant differences; a *P* value of less than 0.05 was considered to indicate statistical significance.

## 3 Results

### 3.1 Generation and characterization of recombinant single-chain HAs

**Figure 1:**
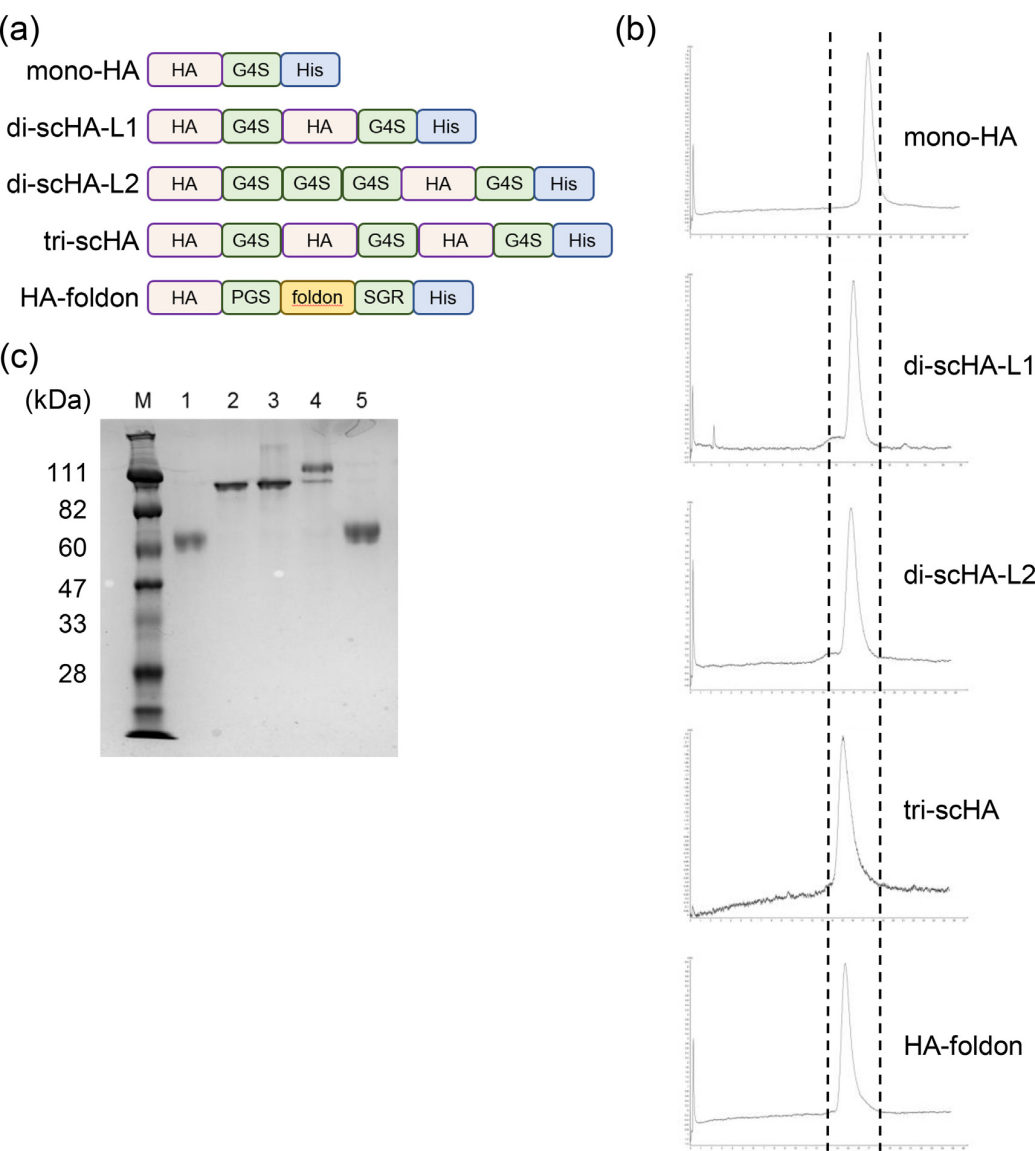
**Construction and characterization of recombinant single-chain HAs**. (a) Schematic primary structures of mono-HA, di-scHA-L1, di-scHA-L2, tri-scHA, and HA-foldon. A His tag was fused to the C-terminus of each HA. G4S indicates the peptide linker GGGGS. (b) Each recombinant HA generated in Expi293F cells was purified by size-exclusion chromatography after metal ion chromatography. (c) Purified recombinant HAs were analyzed through SDS-PAGE followed by staining with Coomassie Brilliant Blue. M, marker; lane 1, mono-HA; lane 2, di-scHA-L1; lane 3, di-scHA-L2; lane 4, tri-scHA; lane 5, HA-foldon. The complete, non-adjusted image is provided as Supplementary Figure S1.

In this study, we designed scHAs that joined two or three monomeric HAs via short peptide linkers (Figure 1a). In particular, we used the ectodomain of HA from H1N1 influenza A viruses (strain: A/California/7/2009 [Cal7]) for generating the recombinant HA, and a His-tag was introduced at the C-terminus to facilitate purification. Dimeric scHAs carried two linker sequences—GGGGS (G4S) for di-scHA-L1 and GGGGSGGGGSGGGGS for di-scHA-L2—and trimeric scHA (tri-scHA) contained the peptide linker GGGGS. We used monomeric HA (mono-HA) and trimeric HA with foldon (HA-foldon) as controls. We then engineered the expression vectors for these HAs and transfected them into Expi293F cells. Each recombinant protein was then purified from the culture supernatants by using immobilized metal ion and size-exclusion chromatography. In size-exclusion chromatography, HA-foldon and tri-scHA eluted at almost the same volume that was expected from their anticipated molecular weight (Figure 1b); in contrast, the elution times of mono-HA, di-scHA-L1, and di-scHA-L2 were longer than those of HA-foldon and tri-scHA (Figure 1b), and the elution times of di-scHA-L1 and di-scHA-L2 were shorter than that of mono-HA (Figure 1b). SDS-PAGE performed under reducing conditions showed that both mono-HA and HA-foldon migrated as a single band with a molecular mass of about 60 kDa (Figure 1c). The molecular weight of HA-foldon was slightly higher than that of mono-HA due to the size of foldon. In contrast, di-scHA-L1, di-scHA-L2, and tri-scHA ran as molecular masses of about 111, 111, and more than 111 kDa, respectively (Figure 1c). In addition to the major band exceeding 111 kDa, tri-scHA showed a minor band at approximately 111 kDa. These data confirm that scHAs are single polypeptides that comprise two or three monomers that are joined through the incorporation of short linker peptides.

### 3.2 Potential of scHAs as vaccine antigens for influenza vaccine

**Figure 2:**
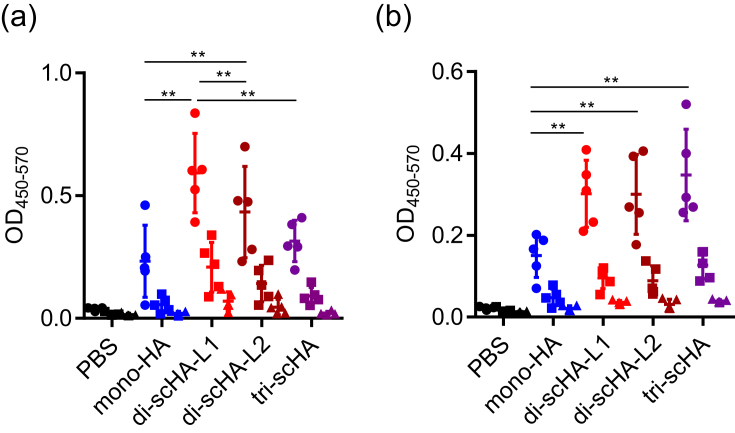
**Potential of scHAs as vaccine antigens for influenza vaccine**. Mice were immunized subcutaneously with mono-HA, di-scHA-L1, di-scHA-L2, or tri-scHA (1 μg/mouse) plus alum. Plasma levels of monomer-HA-specific IgG (a) and trimer-HA-specific IgG (b) were evaluated by using ELISA at 7 days after final immunization; we used 160- (●), 800- (■), and 4,000- (▲) fold dilutions of plasma. Each experiment was performed twice. Data are means ± SD (*n* = 5). Significant differences were analyzed for the 160-fold-diluted plasma samples only. ∗∗, *P* < 0.01 as indicated by Tukey's test.

To assess these scHAs as vaccine antigens in vivo, we immunized mice with each scHA by using aluminum salts (alum) as an adjuvant; alum is widely used as an adjuvant for many important human vaccines, including the diphtheria–tetanus–pertussis inactivated poliomyelitis vaccine, the pneumococcal conjugate vaccine, and hepatitis B vaccine [12]. After the last immunization, we used ELISA to analyze the levels of mono-HA- and HA-foldon–specific IgG in plasma (Figure 2a, b). We describe IgG specific for mono-HA as ‘monomer-HA–specific IgG’ and those specific for HA-foldon as ‘trimer-HA–specific IgG’. Immunization with di-scHA-L1 or di-scHA-L2 induced significantly higher levels of monomer-HA– and trimer-HA–specific IgG than did mono-HA (Figure 2a, b), and the plasma level of monomer-HA–specific IgG in di-scHA-L1–immunized mice was significantly higher than that in di-scHA-L2–immunized mice (Figure 2a). In addition, mice immunized with tri-scHA had significantly higher levels of trimer-HA–specific IgG than those with mono-HA (Figure 2b), but monomer-HA–specific IgG levels did not differ significantly between mono-HA–immunized and tri-scHA–immunized mice (Figure 2a). These results suggest that scHAs—particularly di-scHA-L1—enhanced the antigenicity of mono-HA.

### 3.3 Comparison of antigenicity of dimeric scHA and HA-foldon

**Figure 3:**
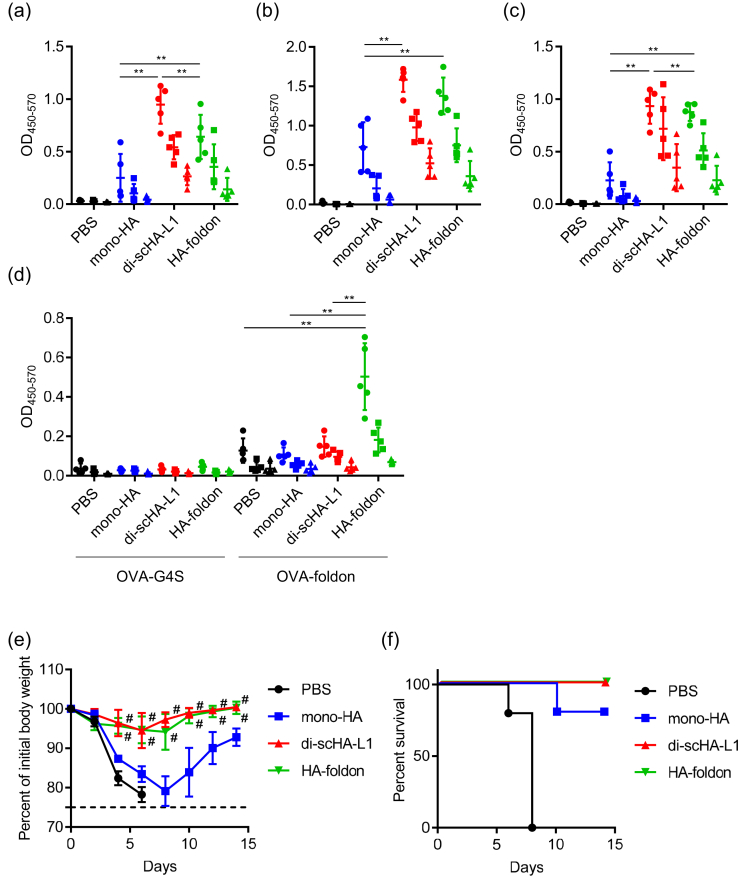
**Comparison of di-scHA-L1 and HA-foldon as antigens for influenza vaccines**. Mice were immunized subcutaneously with mono-HA, di-scHA-L1, or HA-foldon (1 μg/mouse) plus alum. Plasma levels of IgG specific for monomer HA (a), trimer HA (b), Cal7 (c), ovalbumin–foldon (d) and ovalbumin–G4S linker (d) were evaluated by using ELISA at 7 days after final immunization; we used 160- (●), 800- (■), and 4,000- (▲) fold dilutions of plasma. At 10 days after the final immunization, mice were challenged with Cal7. Percentage change in initial body weight (e) and survival rate (f) were monitored after challenge with Cal7. Each experiment was performed twice. Data are means ± SD (*n* = 5). Significance of differences was analyzed for the 160-fold-diluted plasma samples only (a–d); ∗∗, *P* < 0.01 as indicated by Tukey's test. (e) ^#^, *P* < 0.01 vs. group immunized with mono-HA, as indicated by Tukey's test.

To compare the antigenicity of di-scHA-L1 and HA-foldon in vivo, mice were immunized with mono-HA, di-scHA-L1, or HA-foldon by using alum as an adjuvant. The levels of monomer-HA–, trimer-HA–, and Cal7–specific IgG in the plasma of di-scHA-L1- and HA-foldon–immunized mice were significantly higher than that in mono-HA–immunized mice (Figure 3a–c). In addition, the amounts of monomer-HA– and Cal7–specific IgG in di-scHA-L1–immunized mice were significantly greater than that in HA-foldon–immunized mice (Figure 3a, c), but trimer-HA–specific IgG quantities did not differ between di-scHA-L1– and HA-foldon–immunized mice (Figure 3b). Next we used recombinant ovalbumins that contained foldon or G4S linker to examine the levels of foldon-specific and G4S-linker–specific IgG. The level of IgG specific for foldon-containing ovalbumin was significantly higher in HA-foldon–immunized mice than that in mono-HA– or di-scHA-L1–immunized mice (Figure 3d). In contrast, we did not observe any induction of IgG specific for ovalbumin containing G4S linker in either HA-foldon–immunized or di-scHA-L1–immunized mice (Figure 3d). After the last immunization, we challenged immunized mice with Cal7 and then assessed their body weights (Figure 3e) and survival rates (Figure 3f). Non-immunized control mice rapidly lost weight, and all mice died within 8 days after Cal7 challenge. In contrast, both di-scHA-L1– and HA-foldon–immunized mice survived without loss of body weight, whereas mono-HA–immunized mice showed rapid body weight loss. In addition, body weight loss did not differ between di-scHA-L1– and HA-foldon–immunized mice. These results suggest that the antigenicity of di-scHA-L1 is as strong as that induced by using the standard HA-foldon.

**Figure 4:**
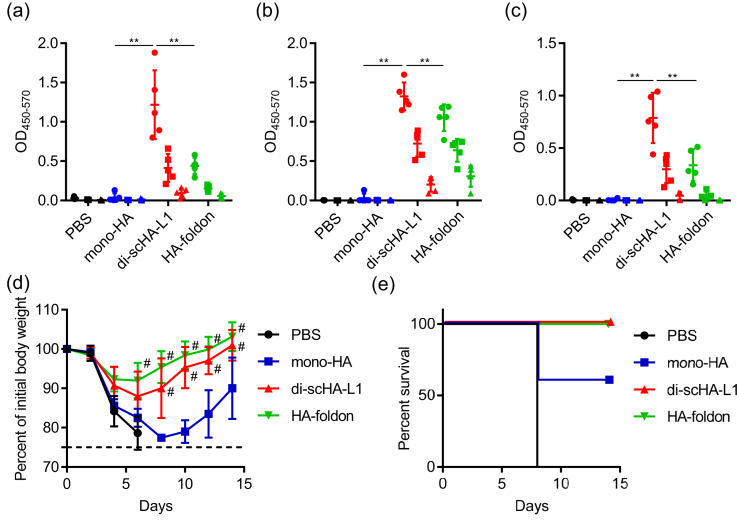
**Vaccine effect of dimeric scHA-L1 plus CpG ODN**. Mice were immunized subcutaneously with mono-HA, di-scHA-L1, or HA-foldon (1 μg/mouse) plus CpG ODN. Plasma levels of monomer-HA-specific IgG (a), trimer-HA-specific IgG (b), and Cal7-specific IgG (c) were evaluated by using ELISA at 7 days after final immunization; we used 160- (●), 800- (■), and 4,000- (▲) fold dilutions of plasma. At 10 days after the final immunization, mice were challenged with Cal7. Percentage change in initial body weight (d) and survival rates (e) were monitored after challenge with Cal7. Each experiment was performed twice. Data are means ± SD (*n* = 5). Significant differences were analyzed for the 160-fold-diluted plasma samples only (a–c); ∗∗, *P* < 0.01 as indicated by Tukey's test. (d) ^#^, *P* < 0.01 vs. group immunized with mono-HA, as indicated by Tukey's test.

We then compared the antigenicity of di-scHA-L1 and HA-foldon when used with another adjuvant, CpG ODN. CpG ODNs are short single-stranded synthetic DNA fragments that contain the immunostimulatory CpG motif and that act as a Toll-like receptor 9 agonist [13]. The levels of monomer-HA-, trimer-HA-, and Cal7-specific IgG in di-scHA-L1–immunized mice were significantly higher than that in not only mono-HA–immunized mice but also HA-foldon–immunized mice (Figure 4a–c). After Cal7 challenge, all di-scHA-L1– and HA-foldon–immunized mice survived without significant loss of body weight, whereas mono-HA–immunized mice showed rapid body weight loss and decreased survival rate (Figure 4d, e). These results suggest that di-scHA-L1 is an effective antigen regardless of the type of adjuvant used.

## 4 Discussion

In the current study, we designed scHAs by using short peptide linkers composed of glycine and serine. Recombinant scHAs had the appropriate predicted molecular weights, indicating lack of aggregation (Figure 1), although the tri-scHA preparation contained minor amounts of an incomplete smaller form in addition to the predominant, full-length form (Figure 1c). scHAs induced stronger antibody responses than mono-HA (Figures 2 and 3). Because both di-scHA-L1 and di-scHA-L2 induced strong HA-specific antibody responses (Figure 2), we speculate that difference in the linker length of these two constructs has little effect on the structure of scHA and its potential as an antigen. Furthermore, scHAs, particularly di-scHA-L1, induced HA-specific antibody responses as strong as those generated when HA-foldon was used as an antigen and effectively protected against virus challenge (Figures 3 and 4). It is interesting that dimeric scHA is sufficient to induce a vaccine effect even though native HA forms a trimeric structure. In general, compared with monomeric antigens, multimerization of antigen enhances B-cell receptor crosslinking, which is followed by strong B cells activation and an increased antigen-specific antibody response. In fact, HA-foldon enhances the antibody response to mono-HA [7, 10]. Further investigation is needed to clarify whether dimeric scHA and trimeric HA induce similarly extensive B-cell receptor crosslinking.

G4S linkers similar to those we used in our scHAs have already been applied extensively for generating recombinant single-chain antibody fragment (scFv) [14]. The antigenicity of scFv has been assessed clinically in humans and is very low, suggesting that multimerization of antigen by using similar short linkers is a rational approach for generating recombinant proteins that lack the trimerization domains. In fact, we here showed that di-scHA-L1 does not lead to G4S-linker–specific IgG, whereas HA-foldon induces foldon-specific IgG; these findings support the low immunogenicity of G4S linkers (Figure 3d).

Here, we used alum as a representative Th2-inducing adjuvant and CpG ODN as a representative Th1-inducing adjuvant. In contrast to alum, CpG ODN induces both Th1-type immune responses and the activation of CD8^+^ cytotoxic T lymphocytes [13]; these are important properties for maximizing vaccine efficacy against various infectious diseases, including influenza virus and hepatitis C [15]. Therefore CpG ODN is likely to be used as a vaccine adjuvant against infectious diseases [13] and, in fact, has already been approved for clinical use as a hepatitis B virus vaccine adjuvant [16]. In our current study, immunization with di-scHA-L1 induced strong antibody responses when used with either alum or CpG ODN as adjuvant. In particular, di-scHA-L1 induced significantly higher antibody responses than HA-foldon when we used CpG ODN as an adjuvant, whereas the preventive effects against influenza virus were the same (Figure 4). Several types of CpG ODNs that differ in structure, physical features, and immunostimulatory properties are available [13, 17]. In the current study, we used a B-type CpG ODN, which has synthetic phosphorothioate backbone [13, 17]. Phosphorothioated DNA is more resistant to DNase than natural phosphodiester DNA [17]. In addition, phosphorothioate ODNs bind nonspecifically to proteins [18]. We speculate that the binding strength of CpG ODN to di-scHA-L1 might differ from that to HA-foldon, resulting in the difference in antibody responses between CpG ODN adjuvanted di-scHA-L1 and CpG ODN adjuvanted HA-foldon.

Here we used subcutaneous injection as the administration route and HA from H1N1 influenza virus as an antigen. Because current influenza vaccines are administered through both intramuscular and intranasal routes [19, 20], it is essential to evaluate the potential of intranasally administered di-scHA-L1. In addition, the strategy we followed in the current study likely could easily be applied to recombinant HAs from other influenza virus strains, including H5N1 and H7N9. This broad applicability supports the rapid production of recombinant HAs as antigens when a pandemic strain emerges. Furthermore, recent studies have shown that cross-reactive antibodies against highly conserved conformational epitopes in the stem region of HA contribute to cross-protection against heterologous strains [2]. To enhance antibody responses against the stem region of HA, various type of recombinant HAs—including headless, chimeric, and hyperglycosylated recombinant HAs—have been developed [21, 22, 23, 24]. Because these other recombinant HAs still contain trimerization domains, the strategy we show here likely would apply to them as well. In addition, we consider that our approach is a versatile method for generating other recombinant multimeric antigens for vaccines, such as recombinant envelope proteins of human immunodeficiency virus [25, 26, 27] and recombinant fusion glycoprotein of respiratory syncytial virus [28, 29]. Therefore, we believe that single-chain antigens might present new avenues for recombinant antigen-based vaccines.

## Declarations

### Author contribution statement

Atsushi Kawai: Conceived and designed the experiments; Performed the experiments; Analyzed and interpreted the data; Wrote the paper.

Yasuyuki Yamamoto: Conceived and designed the experiments; Performed the experiments; Analyzed and interpreted the data.

Yasuo Yoshioka: Conceived and designed the experiments; Analyzed and interpreted the data; Wrote the paper.

### Funding statement

This work was supported by grants from the 10.13039/501100001691Japan Society for the Promotion of Science (10.13039/501100001691JSPS KAKENHI grant numbers JP17H04009 and JP18K19401 to Y. Yoshioka).

### Competing interest statement

The authors declare the following conflict of interests: Y. Yamamoto and Y. Yoshioka; employed by The Research Foundation for Microbial Diseases of Osaka University.

### Additional information

No additional information is available for this paper.
