# Supplementary figures and images for: Vaccine effect of recombinant single-chain hemagglutinin protein as an antigen
Source: Heliyon. 2020 Jun 27;6(6):e04301. doi: 10.1016/j.heliyon.2020.e04301 (PMC7327749; doi:10.1016/j.heliyon.2020.e04301)

## Slide 1
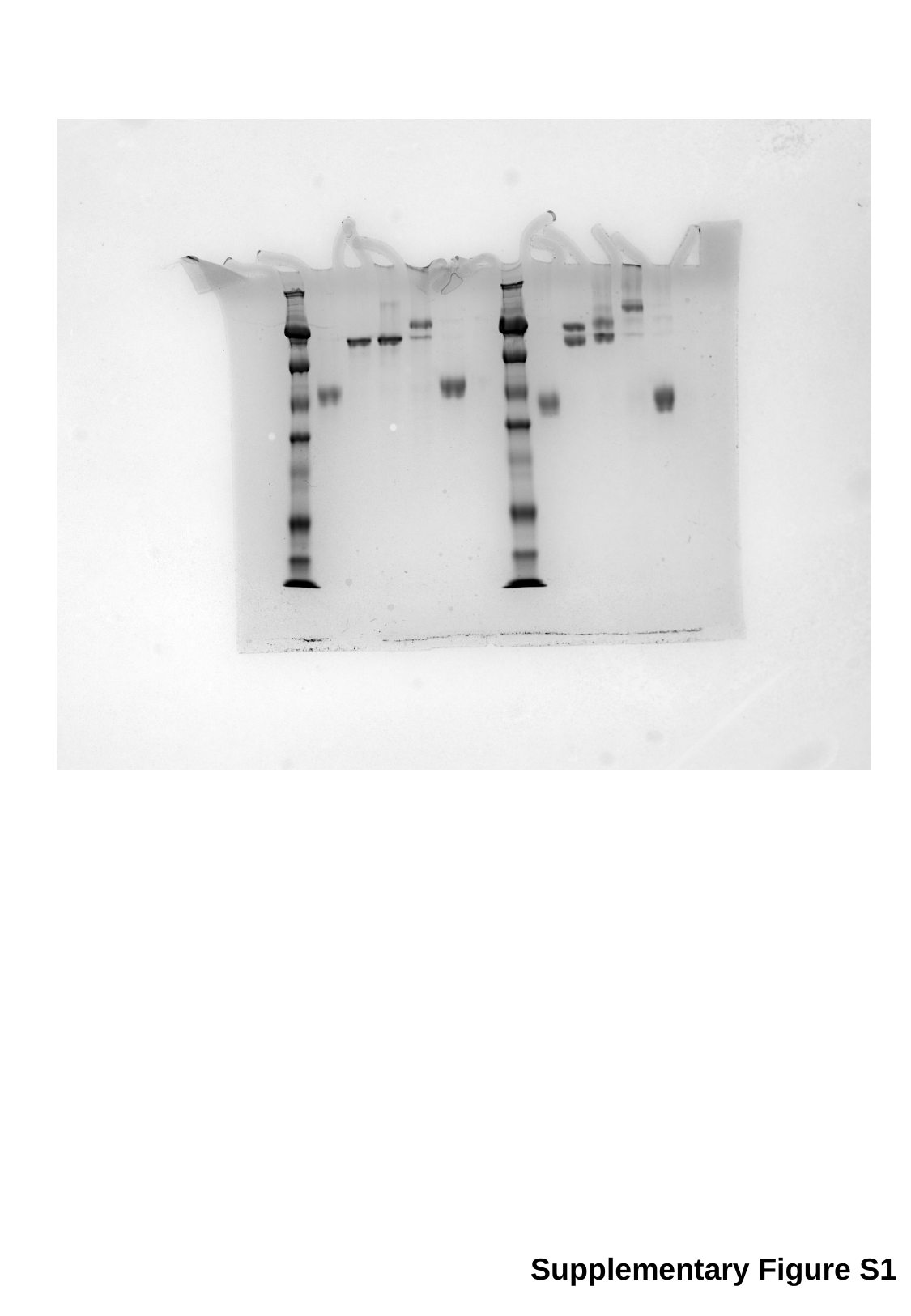

Supplementary Figure S1

Supplement: Supplementary file 1 — Supplementary Figure S1 [file mmc1.pptx]
